# MicroRNA response to hypoxic stress in soft tissue sarcoma cells: microRNA mediated regulation of HIF3α

**DOI:** 10.1186/1471-2407-14-429

**Published:** 2014-06-13

**Authors:** Caroline MM Gits, Patricia F van Kuijk, Jonneke CWM de Rijck, Nikky Muskens, Moniek BE Jonkers, Wilfred F van IJcken, Ron HJ Mathijssen, Jaap Verweij, Stefan Sleijfer, Erik AC Wiemer

**Affiliations:** 1 Department of Medical Oncology, Erasmus University Medical Center – Erasmus MC Cancer Institute, Rotterdam, the Netherlands; 2 Center for Biomics, Erasmus University Medical Center, Rotterdam, the Netherlands; 3 Department of Medical Oncology, Erasmus MC Cancer Institute, Erasmus University Medical Center, Rm Be422, Wytemaweg 80, 3015 CN Rotterdam, the Netherlands

**Keywords:** miRNA, Hypoxia, HIF3α, Soft tissue sarcomas, miR-210-3p, miR-485-5p

## Abstract

**Background:**

Hypoxia is often encountered in solid tumors and known to contribute to aggressive tumor behavior, radiation- and chemotherapy resistance resulting in a poor prognosis for the cancer patient. MicroRNAs (miRNAs) play a role in the regulation of the tumor cell response to hypoxia, however, not much is known about the involvement of miRNAs in hypoxic signalling pathways in soft tissue sarcomas (STS).

**Method:**

A panel of twelve STS cell lines was exposed to atmospheric oxygen concentrations (normoxia) or 1% oxygen (hypoxia) for up to 48 h. Hypoxic conditions were verified and miRNA expression profiles were assessed by LNA™ oligonucleotide microarrays and RT-PCR after 24 h. The expression of target genes regulated by hypoxia responsive miRNAs is examined by end-point PCR and validated by luciferase reporter constructs.

**Results:**

Exposure of STS cell lines to hypoxic conditions gave rise to upregulation of Hypoxia Inducible Factor (HIF) 1α protein levels and increased mRNA expression of HIF1 target genes CA9 and VEGFA. Deregulation of miRNA expression after 24 h of hypoxia was observed. The most differentially expressed miRNAs (p < 0.001) in response to hypoxia were miR-185-3p, miR-485-5p, miR-216a-5p (upregulated) and miR-625-5p (downregulated). The well-known hypoxia responsive miR-210-3p could not be reliably detected by the microarray platform most likely for technical reasons, however, its upregulation upon hypoxic stress was apparent by qPCR. Target prediction algorithms identified 11 potential binding sites for miR-485-5p and a single putative miR-210-3p binding site in the 3’UTR of HIF3α, the least studied member of the HIF family. We showed that HIF3α transcripts, expressing a 3’UTR containing the miR-485-5p and miR-210-3p target sites, are expressed in all sarcoma cell lines and upregulated upon hypoxia. Additionally, luciferase reporter constructs containing the 3’UTR of HIF3α were used to demonstrate regulation of HIF3α by miR-210-3p and miR-485-5p.

**Conclusion:**

Here we provide evidence for the miRNA mediated regulation of HIF3α by hypoxia responsive miRNAs in STS, which may help to tightly regulate and fine-tune the hypoxic response. This provides a better insight into the mechanisms underlying the hypoxic response in STS and may ultimately yield information on novel prognostic and predictive markers or targets for treatment.

## Background

Hypoxia is a condition of reduced oxygen tension that is often encountered in solid tumors including soft tissue sarcomas (STS) when they outgrow their blood supply. The tumor cells respond to the hypoxic conditions by inducing genes that regulate various biological processes including cellular proliferation, apoptosis, metabolism, angiogenesis and migration [1]. A key element in the response to hypoxia is the upregulation of HIF1, a member of the family of hypoxia inducible factors (HIFs) also comprising HIF2 and HIF3 [2]. These transcription factors consist of a tightly controlled alpha (HIFα), and a constitutively activated beta subunit (HIFβ or ARNT family). Upon hypoxia, the alpha subunit is stabilized, translocated to the nucleus, where it dimerizes to the beta subunit. The HIF complex binds to hypoxia responsive elements (HREs) in gene promoters, thereby inducing the transcription of genes important for the adaption to low oxygen concentrations. HIF1α and HIF2α have similar protein domain structures, are regulated in a similar fashion and share several target genes [3], although their expression patterns differ in some tissues and some unique target genes have been identified [4]. The function(s) of HIF3α and the way it is regulated are still largely unknown. Complicating the study of HIF3α is the fact that alternative splice variants are recognized coding for at least six isoforms, with structural and functional differences [5-7]. HIF3α is regulated by HIF1 at the transcriptional level and exerts an inhibitory effect on HIF1α or HIF-dependent gene regulation, in a cell-type specific manner [5-11].

Tumor hypoxia and the accompanying biochemical and cellular changes in the tumor cells contribute to aggressive tumor behavior, and radiation- and chemotherapy resistance in many tumor types, including STS [12,13]. STS is a group of relatively rare tumors of mesenchymal origin in which currently more than 50 different histological subtypes are recognized. STS have been reported to present with hypoxic areas resulting in increased tumor proliferation, distant metastases and shorter overall disease survival [14,15]. Given the poor prognosis STS patients face with, a median overall survival of only 1 year for patients presenting with metastatic disease, more insight is needed into the effects of hypoxia in STS and how hypoxia is regulated in STS.

MicroRNAs (miRNAs) are small (~23 nt) non-protein coding RNA molecules that negatively regulate gene expression. MiRNAs play essential roles in a wide variety of cellular processes, such as differentiation, cell cycle regulation, metabolism, apoptosis and stem cell maintenance and are associated with various diseases including cancer [16]. Most cancers display a deregulated miRNA expression profile when compared to relevant normal tissues. Some miRNAs may function as an oncomiR, having either a tumor suppressive or an oncogenic role [17-20]. MiRNA expression can be influenced by changes in the tumor microenvironment [21,22] including conditions like tumor hypoxia [23-26]. The miRNA response to hypoxia, which is often cell-type specific, suggests miRNAs are intimately involved in the cellular reaction to hypoxia [23,24,26] and some are in turn involved in feedback mechanisms that regulate HIF [27-32]. Interestingly, many hypoxia responsive miRNAs (HRMs) are also upregulated in cancer suggesting they also function in tumorigenesis and/or tumor progression [26]. As opposed to the role of HRMs in the major cancer types relatively little is known about the role of hypoxic miRNAs in STS. In this study, we describe the miRNA response to hypoxic stress in sarcoma cell lines revealing the involvement of miRNAs in the hypoxic signalling in STS.

## Methods

### Cell culture

A panel of 12 soft tissue sarcoma cell lines was used, consisting of the fibrosarcoma cell lines HT1080, SW684 and BG-8, the liposarcoma cell line SW872, the leiomyosarcoma cell lines SK-UT-1 and SK-LMS-1, the synovial sarcoma cell line SW982 and the rhabdomyosarcoma cell lines RH30, A204, A673, RD and SJCRH30. Note that the A673 (ATCC®CRL-1598) cell line is now listed on the ATCC website as Ewing sarcoma. The myxoid fibrosarcoma cell line BG-8 was a kind gift from Dr. A. Carnero (Centro Nacional de Investigaciones Oncológicas, Madrid, Spain); HT1080, SK-UT-1, RD and RH-30 were acquired from Dr. M. Debiec-Rychter (KU Leuven, Leuven, Belgium) and SJCRH30 was obtained from Dr. L. Alberti (Université de Lyon, Centre Léon Bérard, Lyon, France). All further cell lines were obtained from the ATCC, Rockville, MD, USA. The cell lines were cultured in RPMI 1640/GlutaMAX (Invitrogen, Bleiswijk, The Netherlands) supplemented with 10% fetal bovine serum, at 37°C, 5% CO_2_.

### Hypoxic experiments

The 12 cell lines comprising the soft tissue sarcoma panel were cultured under standard conditions and seeded in 6 well culture plates or 9 cm cell culture dishes (miRNA profiling experiment) in duplicate at such a cell density that 50% confluency was reached after 48 h. Subsequently half of the culture plates was kept under standard, normoxic (21% O_2_) culture conditions whereas the other half was transferred to a hypoxic incubator and cultured at 37°C, 5% CO_2_, 1% O_2_ for 6 h to 48 h as indicated. After the appropriate normoxic/hypoxic incubation period cells were rapidly washed with ice-cold PBS after which total RNA was isolated (three separate wells/9 cm dish) and total protein lysates (three separate wells) were prepared.

### RNA isolation

Total RNA was isolated using RNAbee (Tel Test Inc., Friendswood, TX, USA) according to the manufacturer’s recommendation. The RNA concentration and quality were determined on a Nanodrop-1000 (Nanodrop Technologies, Wilmington, DE, USA).

### MiRNA microarray

MiRNA profiling was performed essentially as previously described [33]. In brief, 1 μg total RNA was fluorescently labeled with Cy3 using the ULS™ aRNA Labeling Kit (Kreatech Diagnostics, Amsterdam, The Netherlands). Labeled RNA was hybridized with locked nucleic acid (LNA™) modified oligonucleotide capture probes (Exiqon) spotted in duplicate on Nexterion E slides. The capture probe set (based on miRBase version 10, annotation version 13) contains 1344 probes of which 725 are capable of detecting human miRNAs. Hybridized slides were scanned and median spot intensity was determined using ImaGene software (BioDiscovery Inc., Hawthorne, CA, USA). After background subtraction, expression values were quantile normalized using R software (http://cran.r-project.org; version 2.15.2, release oct. 2012), low expressed miRNAs and obvious outliers/bad spots were removed, and duplicate spots were averaged. Outliers/bad spots were identified by determining the average expression value of each miRNA and examining whether duplicate spots differ from the average (>2 × greater or >5 × smaller). Flagged spots were checked by eye to determine why the signal is aberrant e.g. a speck of dust or printing error. Only in case of an apparent technical error, noted in less than 1% of the spots, a spot was discarded. For each expression value, the ratio to the geometric mean of the samples was log2 transformed. These values were used to determine differentially expressed miRNAs. Hierarchical clustering analyses were performed in Spotfire (Spotfire DecisionSite 9.1, Tibco Software, Somerville, MA. USA).

### qPCR analysis of miRNA

MiR-210-3p expression was examined using TaqMan® MicroRNA Assays (Applied Biosystems, Nieuwerkerk aan den IJssel, The Netherlands). In brief, total RNA (50 ng) was reverse transcribed using specific miRNA primers and the TaqMan® MicroRNA Reverse Transcription Kit (Applied Biosystems). The resulting cDNA was used as input in a quantitative real-time PCR (qPCR) using the miRNA specific primer/probe mix together with the TaqMan® Universal PCR Master Mix No AmpErase® UNG (Applied Biosystems) according to manufacturer’s protocol. qPCR data were analyzed with SDS software (version 2.4, Applied Biosystems). MiRNA expression was normalized using RNU43 expression and the comparative C_T_-method [34].

### cDNA-synthesis

RNA (1 μg) was reversed transcribed using the High Capacity cDNA Reverse Transcription Kit (Applied Biosystems) according to the manufacturer’s protocol. The resulting cDNA was used to perform qPCR and end-point PCR analysis.

### qPCR analysis of mRNAs

cDNA (45 ng) was used to perform qPCR using the primer/probe mix from the TaqMan® Gene Expression Assays of human VEGFA (assay ID Hs00900055_m1) and CA9 (assay ID Hs00154208_m1) with exon spanning probes, and TaqMan® Universal PCR Master Mix using the 7500 Fast Real-Time PCR system (all Applied Biosystems) according to the manufacturer’s protocol. HPRT was used as a housekeeper gene for normalization purposes using the comparative C_T_-method. qPCR data was analyzed with SDS software (Applied Biosystems).

### End-point PCR analysis

cDNA (50 ng) was used to perform endpoint PCR using 1 × Green Go Taq Flexi Buffer, 1.25U Go Taq Flexi polymerase, 200 μM dNTP mix, 1.5 mM MgCl_2_ and 300 nM forward and reverse primer of HIF3α (Additional file 1: Table S1a) on a thermal cycler using the following PCR-program: 2 min 95°C, 30-40 cycles of (45 sec 95°C, 45 sec 60-65°C, 45 sec 72°C), 5 min 72°C. PCR-products were analyzed on a 1.5% agarose gel in 0.5 × TBE buffer with 0.5 μg/ml ethidium bromide.

### Protein extraction

Cells were harvested in MCB lysis buffer (50 mM Tris–HCl pH 7.5, 50 mM NaCl, 10% glycerol, 1% NP-40, 0.5% Na-deoxycholate, 20 mM NaF) supplemented with a cocktail of protease and phosphatase inhibitors. Lysates were thoroughly vortexed and further lysed by two subsequent freeze-thaw cycles using liquid nitrogen. Cell debris was spun down and protein concentration was determined by a Bradford assay (BioRad, Veenendaal, The Netherlands).

### Western blotting

Twenty μg of total protein was subjected to SDS-PAGE. Proteins were transferred to a PVDF membrane followed by blocking of the membrane in 5% non-fat dry milk in PBS-Tween (Phosphate buffered saline, 0.05% (v/v) Tween 20) to prevent non-specific antibody binding. Primary and secondary antibody incubations were carried out in the same buffer using anti-HIF1α (610958, mouse monoclonal, 1:500, BD Biosciences, Breda, The Netherlands), anti-HIF3α (ab10134, rabbit polyclonal, 1:500, Abcam, Cambridge, UK) and anti-β-Actin (AC-15, mouse monoclonal, 1:5000, Sigma-Aldrich, Zwijndrecht, The Netherlands) as a loading control. As secondary antibody HRP-conjugated goat-anti-mouse (1:10000, Santa Cruz Biotechnology, Heidelberg, Germany) or goat-anti-rabbit (1:10000, Jackson Immunoresearch, Suffolk, UK) antibodies were used. Antibody incubations were followed by enhanced chemoluminescence (Supersignal West Pico Chemiluminescent Substrate, Thermo Scientific) and visualized on film (Amersham Hyperfilm ECL, GE Healthcare, Diegem, Belgium).

### Mimic transfections

MiRIDIAN microRNA mimics (Thermo Scientific, Etten-Leur, Netherlands) of hsa-miR-210-3p and hsa-miR-485-5p and miRIDIAN microRNA Mimic Negative Control #1 (Thermo Scientific) were transfected in a final concentration of 50 nM using DharmaFECT1 transfection reagent (Thermo Scientific) 24 h after cell seeding. Transfection efficiency was optimized to >95% using fluorescent mimics.

### Cloning

Fragments of the 3’UTR of *HIF3α* (HIF3α-short: 817 bp fragment, HIF3α-long: 3807 bp fragment) were PCR amplified from human genomic DNA (Promega, Leiden, The Netherlands) introducing a XhoI (5’-end) and a NotI site (3’-end). The PCR products were cloned in PCR®-Blunt (Invitrogen), followed by a XhoI and NotI restriction and ligation in the psiCHECK™-2 vector (Promega) behind a *Renilla* luciferase gene. The psiCHECK-2 vector also contains a firefly luciferase gene, which was used for normalization. The resulting constructs are psiCHECK2/HIF3α-short and psiCHECK2/HIF3α-long. Site directed mutagenesis (QuickChange II XL, site directed mutagenesis kit, Agilent Technologies, Amsterdam, The Netherlands) was performed to mutate miR-210-3p and miR-485-5p target sites in the 3’UTR of HIF3α in psiCHECK2/HIF3α-short (Additional file 1: Figure S1). Primer sequences used for cloning and mutagenesis are listed in Additional file 1: Table S1b and S1c.

### Luciferase assay

PsiCHECK-2/HIF3α-short and psiCHECK2/HIF3α-long constructs were transfected using Fugene HD transfection reagent (Promega) 24 h after cell seeding, or when mimics were used, 24 h after miRNA mimic transfection, according to recommendations by the manufacturer. After 24 hours the cells were washed with PBS and lysed by Passive Lysis Buffer (Dual Luciferase® Reporter Assay System, Promega) for 30 min on a shaker platform. Lysates were transferred to white 96-wells microplates. Luciferase Assay Reagent II (Dual Luciferase® Reporter Assay System, Promega) was added, immediately followed by quantitation of the firefly luciferase activity on a luminometer (Aspekt Fluoroskan). Subsequently, Stop & Glo® Reagent (Dual Luciferase® Reporter Assay System, Promega) was added to the mixture, immediately followed by quantitation of the *Renilla* luciferase activity. Relative luciferase signals of duplicates were averaged, values (n = 3) were normalized, and average and standard deviations were calculated. Significant differences in luciferase activity were determined by two-sample t-tests.

### Detection of HRE in miRNA promoter regions

To determine whether the putative promoter regions of miR-216a-5p, miR-185-3p and miR-625-5p contain hypoxia responsive elements (HRE) we downloaded 600 bp of 5’ flanking sequences for each of the three miRNA genes from the Ensembl Genome Browser release 72 (http://www.ensembl.org). We scanned the flanking sequences for the presence of consensus HREs (A/GCGTG) and imperfect HREs.

## Results

### Hypoxia responsive miRNAs in soft tissue sarcoma cells

**Figure 1:**
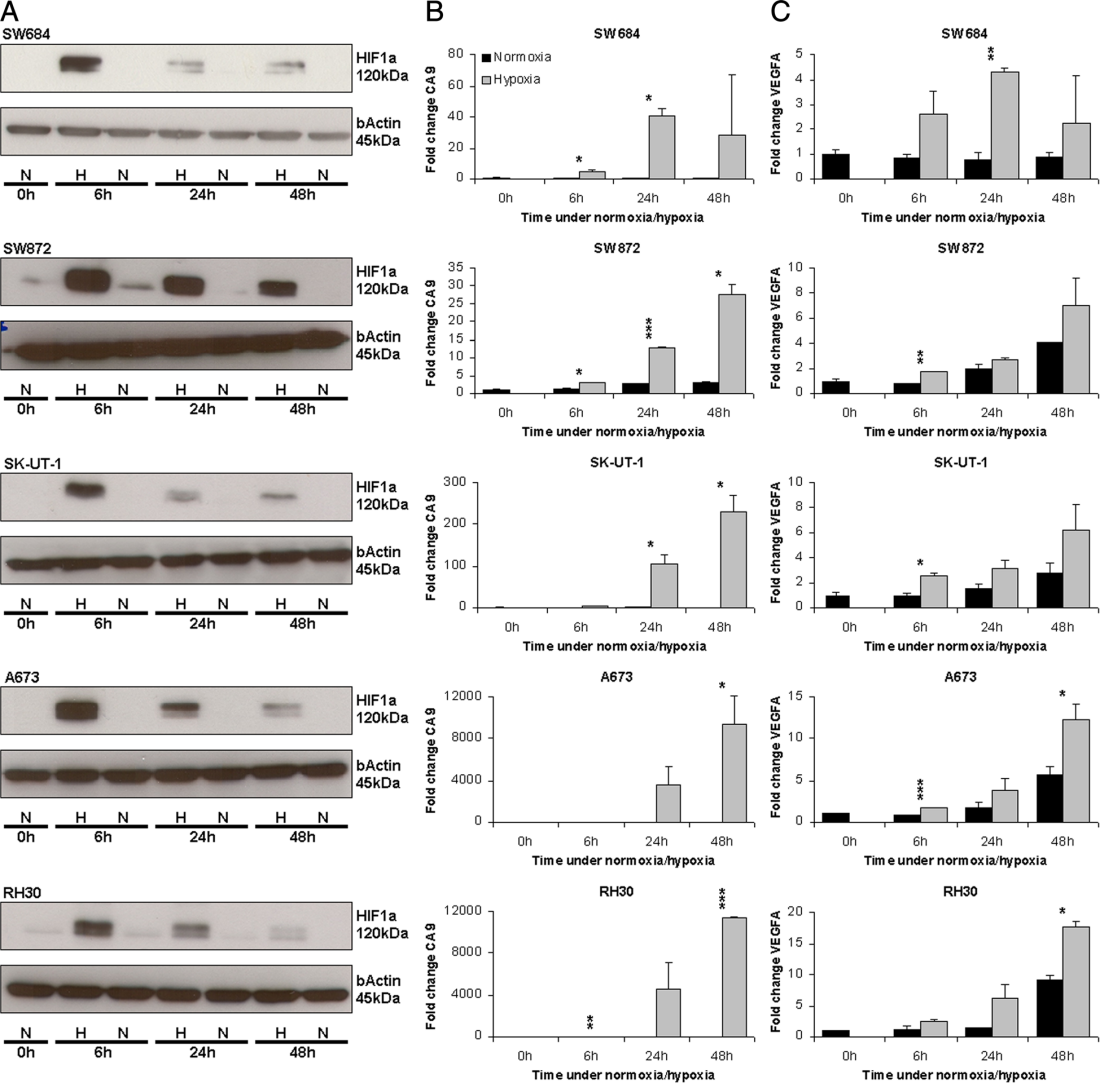
**Hypoxia induces HIF1α protein levels and CA9 and VEGFA transcription in soft tissue sarcoma cell lines. (A)** HIF1α protein levels are stabilized and increase during hypoxia. Cell lines were cultured under hypoxia (H) or normoxia (N), after the indicated times cell lysates were prepared and analysed by Western blotting for HIF1α expression. HIF1α levels peak at 6 hours of hypoxia. Upon prolonged hypoxia (24 and 48 hours) HIF1α protein levels decrease, but remain increased compared to the HIF1α levels detected in cells cultured under normoxic conditions. β-Actin was used as a loading control. **(B,C)** mRNA levels of HIF1 target genes CA9 **(B)** and VEGFA **(C)** are upregulated during hypoxia (grey bars) compared to normoxia (black bars). CA9 and VEGFA expression was determined by RT-PCR and normalized to the expression of HPRT. Bars indicate average fold change of mRNA expression ± S.D.(n = 2) compared to the levels detected at 0 h which are arbitrarily set at 1. Statistical significance between CA9 and VEGF expression in normoxic and hypoxic samples at the various time points was determined by two-sample t-tests: * = p < 0.05, ** = p < 0.005, *** = p < 0.0005. The complete panel of 12 soft tissue sarcoma cell lines was examined, the results obtained with five representative cell lines are shown.

In order to examine the miRNA response to hypoxia in sarcomas, a panel of 12 soft tissue sarcoma cell lines was cultured for 0 h, 6 h, 24 h and 48 h under hypoxic (1% oxygen) or normoxic (21% oxygen) conditions. Exposure to hypoxic conditions was verified by the upregulation of HIF1α protein levels, which is one of the earliest markers of hypoxia. HIF1α protein, being degraded under normoxic conditions, is stabilized during hypoxia. As expected, HIF1α protein levels were significantly increased upon hypoxic stress (Figure 1A). In all 12 cell lines examined peak HIF1α levels were observed at the 6 h time point which decreased after 24 h and 48 h of hypoxia as shown for 5 representative cell lines (Figure 1A). As a consequence of HIF1α upregulation, the transcription of specific HIF1 target genes like CA9 and VEGFA was slightly induced after 6 h and further increased after 24-48 h of hypoxia, as shown for 5 representative cell lines (Figure 1B, C). Note that in particular the increase in CA9 varies considerably between cell lines. The carbonic anhydrase CA9 is a member of a zinc metalloenzyme family that catalyses the reversible hydration of carbon dioxide to carbonic acid [35,36]. CA9 is a transmembrane protein which is found overexpressed in numerous tumor types and induced under hypoxic conditions. Similarly HIF1α stimulates the expression of the vascular endothelial growth factor (VEGFA) which functions as an signal mediating vasculogenesis and angiogenesis [37].

**Figure 2:**
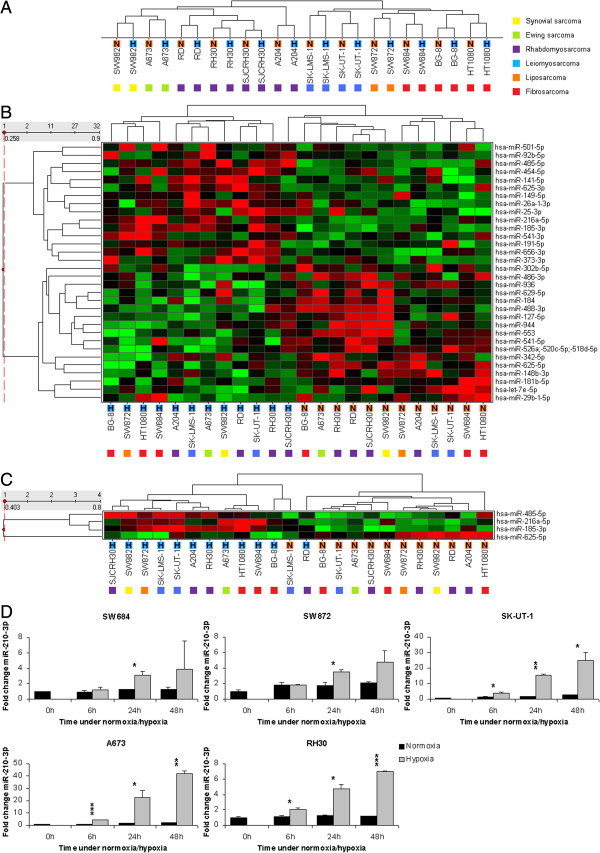
**Hypoxia induces changes in miRNA expression. (A)** Unsupervised hierarchical clustering using miRNA expression data (407 miRNAs) of hypoxic (H) and normoxic (N) cell line samples does not discriminate the hypoxic and normoxic samples. The hypoxic samples cluster together with their normoxic counterparts. **(B)** The most significant differentially expressed miRNAs (two-sample *t*-test, p < 0.05) between cell lines that were cultured under normoxic and hypoxic conditions are used for a supervised hierarchical clustering. The expression of these 32 miRNAs can distinguish hypoxic from normoxic sarcoma cell line samples. **(C)** The four most significant (P < 0.001) differentially expressed miRNAs (miR-485-5p, miR-216a-5p, miR-185-3p and miR-625-5p) discriminate sarcoma cell lines that were cultured under normoxic conditions from those cultured under hypoxic conditions in a supervised hierarchical clustering. **(D)** MiR-210-3p is upregulated during hypoxia. RT-PCR was used to determine miR-210-3p levels in normoxic (black bars) and hypoxic (grey bars) samples from five representative sarcoma cell lines. Expression is normalized against RNU43 expression. Bars indicate average expression fold change ± S.D. (n = 2) of miR-210-3p compared to the expression at 0 h. Statistical significance between expression in normoxic and hypoxic samples at a time point were determined by two-sample t-tests: * = p < 0.05, ** = p < 0.005, *** = p < 0.0005. The different cell lines are indicated by a color code that identifies their tumor of origin: with synovial sarcoma (yellow); Ewing sarcoma (green); rhabdomyosarcoma (purple); leiomyosarcoma (blue); liposarcoma (orange) and fibrosarcoma (red).

Next, the miRNA expression profile was analyzed using microarrays containing LNA™ oligonucleotide capture probes capable of detecting 725 human miRNAs. Since HIF1 target genes were clearly induced after 24 h of hypoxia, miRNA expression levels of cell lines that were cultured for 24 h under normoxic or hypoxic conditions were compared. Unsupervised hierarchical clustering based on the miRNA expression profiles of 407 miRNAs grouped the cell lines on cell type rather than on exposure to hypoxia (Figure 2A), which implies that the changes in miRNA expression due to hypoxia are relatively minor. One major cluster branch contains the rhabdomyosarcoma cell lines and the synovial sarcoma cell line, the other branch contains the other cell lines (leiomyosarcoma, liposarcoma and fibrosarcoma cell lines). Supervised hierarchical clustering using the 32 most differentially expressed miRNAs (p < 0.05; Additional file 1: Table S2) between cell lines exposed to hypoxia or normoxia clearly separated the hypoxic from the normoxic samples (Figure 2B). Fifteen miRNAs were upregulated during hypoxia, while 17 miRNAs were downregulated. The SJCRH30 rhabdomyosarcoma cell line cultures under hypoxia was the only sample that misclustered, which indicates that the hypoxia induced miRNA response in this cell line was apparently weak. The top four differentially expressed miRNAs (p < 0.001) consisted of miR-185-3p, miR-485-5p, miR-216a-5p and miR-625-5p (Figure 2C; Additional file 1: Table S2). Only miR-625-5p was downregulated in most hypoxic samples, whereas the other three miRNAs were predominantly upregulated. MiR-210-3p, a commonly found hypoxia responsive miRNA (HRM), was not reliably detected on our microarray platform. Therefore the miR-210-3p expression in normoxic and hypoxic sarcoma cell line samples was separately determined by qPCR, which revealed a significant upregulation of miR-210-3p in all cell lines after 24 h of hypoxia (Figure 2D).

### HIF3α transcripts are expressed and upregulated under hypoxia in sarcoma cell lines

**Figure 3:**
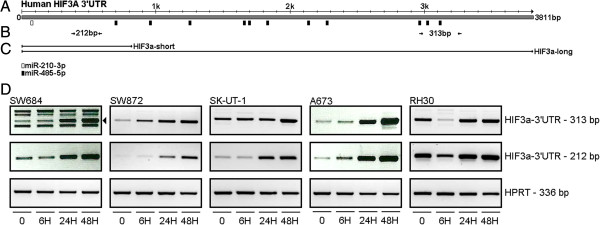
**The 3’UTR of HIF3α contains putative miR-210-3p and miR-485-5p binding sites and is expressed in sarcoma cell lines. (A)** Schematic representation of the 3’UTR of the HIF3α transcript variants 003 and 201 (http://www.ensembl.org, release 72). Depicted are predicted target sites of miR-210-3p (open box) and miR-485-5p (black boxes). **(B)** Arrows indicate location of primers used to detect the presence of HIF3α 3’UTR transcripts. A 212 bp and 313 bp amplification product indicate expression of the 3’UTR of HIF3α transcription variants 003 and 201. **(C)** Lines designate the HIF3α 3’UTR fragments (HIF3α-short, HIF3α-long) which were cloned into the psiCHECK-2 luciferase reporter to verify regulation of HIF3α by miRNAs. **(D)** End-point RT-PCR was used to determine the presence of HIF3α 3’UTR transcripts and their induction upon hypoxia. Depicted are EtBr stained PCR amplification fragments of 212 and 313 bp derived from HIF3α 3’UTR cDNA and an amplified 336 bp HPRT fragment as input control. The results obtained with five representative cell lines are shown.

Candidate target genes for the most differentially expressed miRNAs identified in the microarray analysis were predicted using TargetScanHuman 6.2 (http://www.targetscan.org/) [38]. When we focused on genes known to play a role in the hypoxic response it was noted that miR-485-5p has as much as 11 potential binding sites in the 3’UTR of HIF3α, the less studied member of the family of hypoxia inducible factors. Also miR-210-3p has a single putative binding site in this transcript (Figure 3A). Several human HIF3α transcripts exist due to alternative splicing, coding for different HIF3α isoforms [5-7]. According to the Ensembl Genome Browser release 72 (http://www.ensembl.org) the HIF3α transcript variants 003 and 201 (consistent with HIF3α transcription variant 1, 2 and 3 according to GenBank (http://www.ncbi.nlm.nih.gov/genbank)) contain a long 3’UTR of 3811 bp which includes the potential miR-210-3p and miR-485-5p binding sites. In order to determine whether these transcripts are expressed in our sarcoma cell line panel we used an adapted RT-PCR procedure involving an end-point PCR and specific primers located in the 3’UTR (primer sites are indicated in Figure 3B, primer sequences are listed in Additional file 1: Table S1a). HIF3α transcripts containing a long 3’UTR harboring the mentioned miRNA binding sites could be detected in all cell lines and were upregulated in time under hypoxic conditions (Figure 3D).

### MiR-485-5p and miR-210-3p target HIF3α

**Figure 4:**
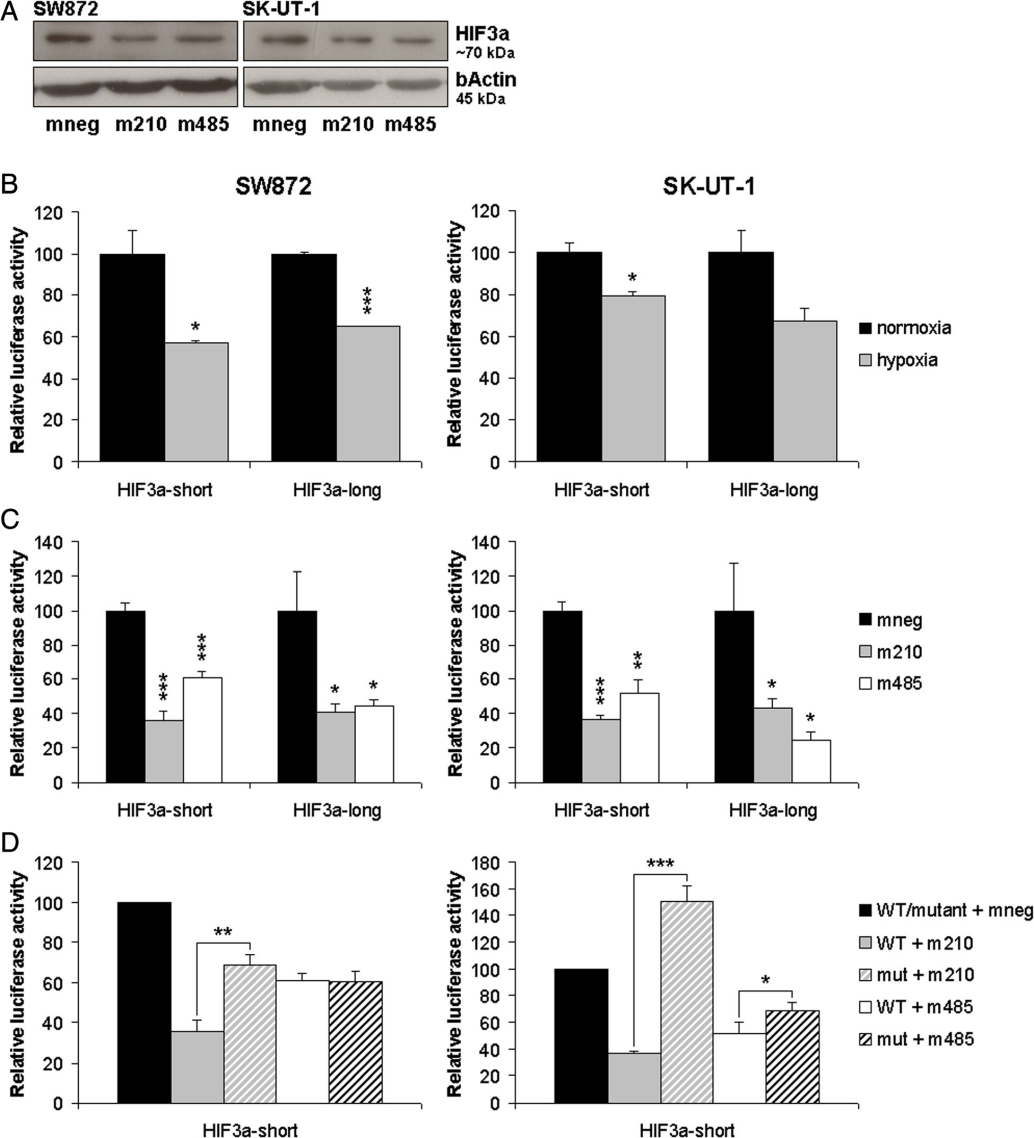
**HIF3α is regulated by miR-210-3p and miR-485-5p. (A)** MiR-210-3p and miR-485-5p mimic overexpression reduce HIF3α protein induction under hypoxic conditions. The sarcoma cell lines SW872 and SK-UT-1 were transfected with mimics of miR-210-3p (m210), miR-485-5p (m485) and a scrambled negative control mimic (mneg). 48 h post-transfection cells were cultured under hypoxic conditions (1% O_2_) for 24 h. Subsequently total protein lysates were prepared and analysed by Western blotting for HIF3α protein expression. β-Actin is used as a loading control. **(B)** Hypoxia responsive miRNAs target HIF3α 3’UTR. SW872 and SK-UT-1 cell lines were transfected with psiCHECK 2 constructs containing short and long 3’UTR fragments of HIF3α. 24 h later the cells were exposed to hypoxia for 24 h. Bars indicate average luciferase activity ± SD (n = 3) measured in hypoxic cell lysates relative to the luciferase activity in normoxic cell lysates which is arbitrarily set at 100. **(C)** MiR-210-3p and miR-485-5p regulate HIF3α. SW872 and SK-UT-1 cell lines were transfected with mimics of miR-210-3p (m210), miR-485-5p (m485) or a scrambled control mimic (mneg) followed after 24 h by a transfection with psiCHECK 2 constructs containing short and long fragments of the HIF3α 3’UTR. Bars indicate average luciferase activity ± SD (n = 3) measured in cell lysates after 24 h. **(D)** MiR-210-3p and miR-485-5p regulate HIF3α. SW872 and SK-UT-1 cell lines were transfected with mimics of miR-210-3p (m210), miR-485-5p (m485) or a scrambled control mimic (mneg) followed, after 24 h, by a transfection with a psiCHECK 2-HIF3α-short construct containing either wild-type (WT) or mutated (mut) miR-210-3p and miR-485-5p binding sites. Bars indicate average luciferase activity ± SD (n = 3) measured in cell lysates. Statistical significance was determined by two-sample t-tests: * = p < 0.05, ** = p < 0.005, *** = p < 0.0005.

The effects of miR-210-3p and miR-485-5p overexpression on HIF3α, protein levels were examined by immunoblotting. The sarcoma cell lines SW872 (liposarcoma) and SK-UT-1 (leiomyosarcoma) were transfected with mimics of miR-210-3p, miR-485-5p or a scrambled negative control. 48 h post-transfection the cells were exposed to hypoxia for 24 h to induce HIF3α. Figure 4A clearly shows that overexpression of the miRNA mimics resulted in a reduced induction of HIF3α when compared to the HIF3α protein levels observed in cells transfected with a scrambled control mimic (mneg). The detected HIF3α protein has a relative molecular weight of approximately 70 kDa which is in line with the predicted molecular weight of the HIF3α proteins encoded by the 003 or 201 HIF3α transcript variants.To demonstrate that during hypoxia HIF3α is regulated by hypoxia responsive miRNAs, a short (HIF3α-short, 817 bp) and a long (HIF3α-long, 3807 bp) fragment of the HIF3α 3’UTR (variants 003/201) (Figure 3C) were cloned in a psiCHECK2 luciferase reporter. The psiCHECK2/HIF3α-short construct encompassed the putative miR-210-3p binding site and the first miR-485-5p binding site. The psiCHECK2/HIF3α-long construct included an additional 10 potential binding sites for miR-485-5p. The SW872 an SK-UT-1 cell lines were transfected with the psiCHECK2/HIF3α-short and psiCHECK2/HIF3α-long constructs. The next day the cells were exposed to hypoxia for 24 h, after which the luciferase activity in cell lysates was determined. Luciferase activity in SW872 hypoxic lysates was reduced to 57% and 65% in the HIF3α-short and HIF3α-long transfectants, respectively, when compared to the levels seen at normoxic circumstances (Figure 4B, left panel). In the hypoxic SK-UT-1 lysates the luciferase activity was decreased to 79% and 67% in the HIF3α-short and HIF3α-long transfectants, respectively (Figure 4B, right panel). These results indicate that during 24 h hypoxia the 3’UTR of HIF3α is targeted, most likely by hypoxia responsive miRNAs, causing a reduction of the luciferase activity.To examine whether downregulation of HIF3α is mediated by miR-210-3p and/or miR-485-5p we transfected miRNA mimics for miR-210-3p and miR-485-5p in SW872 and SK-UT-1 cells, after 24 h followed by the transfection of the psiCHECK2/HIF3α 3’UTR luciferase reporter constructs. Compared to the scrambled negative control (mneg), miR-210-3p overexpression decreased the luciferase activity to 36% and 41% in SW872 (Figure 4C, left panel) and 37% and 43% in SK-UT-1 (Figure 4C, right panel) in the HIF3α-short and HIF3α-long transfectants respectively. MiR-485-5p overexpression reduced the luciferase activity to 61% in the HIF3α-short construct in the SW872 cell line. When ten additional putative binding sites for miR-485-5p were introduced in the HIF3α-long transfectant, the luciferase activity dropped to 45% (Figure 4C, left panel). Overexpression of miR-485-5p decreased luciferase activity to 52% in SK-UT-1 cells transfected with psiCHECK2/HIF3α-short and further reduced luciferase activity to 24% in the HIF3α-long transfectants (Figure 4C, right panel).

To prove whether the regulation of HIF3α is due to direct binding of miR-210-3p and miR-485-5p to target sites in the 3’UTR, the predicted binding sites were mutated to prevent miRNA binding (Additional file 1: Figure S1). Since the regulatory effect of the single miR-485-5p site present in the HIF3α-short construct was greater than the effect of the additional miR-485-5p binding sites present in the HIF3α-long construct, only the miR-485-5p target site in the HIF3α-short construct was mutated. The presence of a mutated miR-210-3p binding site significantly increased (SW872) or restored (SK-UT-1) luciferase activity (Figure 4D). Mutation of the miR-485-5p target site did not affect luciferase activity in SW872 cells and restored luciferase activity to some extent in the SK-UT-1 cells. We conclude that miR-210-3p directly regulates HIF3α whereas the regulation observed with miR-485-5p is primarily due to an indirect effect. However, we cannot rule out that direct regulation of HIF3α does occur mediated by one or more of the additional miR-485-5p binding sites in the HIF3α 3’UTR.

## Discussion

STS, like all solid tumors, can present with hypoxic areas, a state which is associated with disease progression and bad prognosis [14,15]. To date, little is known about the involvement of miRNAs in the hypoxic response of STS. Greither et al. reported that the expression of miR-210-3p, a well-known hypoxia responsive miRNA, is associated with poor survival in STS [39]. We identified 32 miRNAs that are deregulated upon hypoxia in a panel of 12 soft tissue sarcoma cell lines. Particularly, miR-185-3p, miR-485-5p, miR-216a-5p were significantly upregulated, while miR-625-5p was significantly downregulated. In addition we detected induction of miR-210-3p upon hypoxia using a RT-PCR procedure. In all cell lines examined, our array platform did not detect a significant upregulation of miR-210-3p expression upon hypoxia. In fact, expression levels detected were too low to be considered for subsequent analyses. This is most likely due to the fact that the LNA capture probe for miR-210-3p was not optimally designed. Exiqon varies both length and LNA contents of the capture probes to obtain a Tm normalized probe with appropriate affinity for its target miRNA. Although the design and composition of the capture probes are proprietary information we learned that Exiqon has redesigned the miR-210-3p capture probe in subsequent versions of the capture probe set. A subset of the deregulated miRNAs, i.e. miR-185-3p, miR-191, miR-210-3p, miR-373 (upregulated), miR-148b, miR-181b and miR-342-5p (downregulated), was previously identified as hypoxia responsive miRNAs (HRM) in other studies involving different cancer types [40-43]. These miRNAs may be regarded as general responders to hypoxia. However the majority of miRNAs we detected has not been previously associated with hypoxia suggesting a cell-type specific miRNA response, as has been reported before [25,44].

The molecular mechanisms responsible for miRNA deregulation in response to hypoxia are for a large part still unclear as is the precise role of each of the HRM in the hypoxia response. As the key response to hypoxic conditions is the stabilization of HIF1, HRMs can be regulated by HIF-dependent or, alternatively, by HIF-independent mechanisms (reviewed in [44,45]). Some HRMs, such as miR-210-3p, contain hypoxia responsive elements (HREs) in their promoter region and are directly regulated by the HIF1 transcription factor in response to hypoxia [26,46,47]. Although HIF1 is generally known as a transcriptional activator, it can also function as a transcriptional repressor [48], which could account for the downregulated miRNAs under hypoxic conditions. Another HIF-dependent mechanism by which hypoxia responsive miRNAs are regulated is via a HIF1 induced transcription factor, such as TWIST, which induces miR-10b expression [49]. A HIF-independent mechanism involved in the regulation of HRMs is e.g. the induction of Akt2 upon hypoxia, which in turn upregulates miR-21 [50]. Also deregulation of the miRNA biogenesis during hypoxia could contribute to altered miRNA expression levels. Recently it was reported that hypoxia enhanced the association between EGFR and Ago2, resulting in increased Ago2-phosphorylation and reduced binding of Dicer to Ago2, thereby inhibiting miRNA processing [51]. In contrast, in another study hypoxia did not alter the expression of key miRNA machinery proteins (i.e. Drosha, Exp5, Dicer, Ago2 and DP103) in human trophoblasts [52]. In order to determine whether our top hypoxia responsive miRNAs (miR-185-3p; miR-485-5p; miR-216a-5p and miR-625-5p) contain HREs in their putative promoter region we screened 600 bp flanking sequences upstream of the transcription start site of the primary miRNA. With the exception of miR-485-5p of which the gene resides in a densely packed miRNA cluster on chromosome 14, the genes for miR-185-3p, miR-216a-5p and miR-625-5p are not clustered. No consensus HRE (A/GCGTG) could be detected upstream of miR-625-5p (which was downregulated during hypoxia), whereas miR-216a-5p contains a ‘ACGTGC’(position -42 to -37) and miR-185-3p contains a ‘CCGTG’(position -305 to -301). Although the latter HRE does not perfectly match the consensus HRE sequence, imperfect HREs have been found to be functional [11,53,54]. Therefore it is possible that miR-216a-5p and miR-185-3p are regulated by HIF1.

Since miR-210-3p is considered a general responder to hypoxia irrespective of cell-type and exact hypoxic conditions, its function is thought to be universal. MiR-210-3p has been demonstrated to have a role in e.g. cell proliferation, angiogenesis, apoptosis, DNA repair and mitochondrial metabolism (reviewed in [55-57]). The function of other, less commonly deregulated HRMs is poorly understood, but these miRNAs are likely to have a cell-type specific function. Not much is known about miR-485-5p. This miRNA was downregulated in ependymomas [58] and suppressed dendritic spine development in rats [59]. Furthermore miR-485-5p was downregulated in Alzheimers disease [60] and malignant serous ovarian tumors, and correlated with FIGO grade in the latter [61]. Computational analysis predicted as much as eleven putative miR-485-5p binding sites in the 3’UTR of HIF3α. Also miR-210-3p has a potential binding site in the 3’UTR of this member of the family of hypoxia inducible factors. Note that there are only two HIF3α transcripts (variants 003 and 201) listed (http://www.ensembl.org; release 72) that habor a 3’UTR long enough to encompass all miR-485-5p binding sites in addition to the miR-210-3p site. At least two other HIF3α transcripts that contain a smaller 3’UTR (478 bp in variant 006 and 287 bp in variant 007) also contain the miR-210-3p binding site suggesting that the HIF3α isoforms they encode may be controlled by miR-210-3p. We demonstrated that HIF3α is regulated by miR-210-3p and in an indirect fashion by miR-485-5p. We also showed that HIF3α mRNA levels are induced upon hypoxia as expected of a hypoxia inducible factor. Under the same conditions, however, the expression of hypoxia responsive miRNAs like miR-210-3p and miR-485-5p is upregulated that affect HIF3α protein expression. We postulate that miRNAs help to fine-tune HIF3α protein expression and in this way regulate the cellular hypoxic response. They can do so by affecting the translation of specific HIF3α transcripts, however, we cannot rule out that part of the regulation takes place via HIF3α mRNA degradation. Although HRMs targeting HIF3α has never been documented before, miRNA regulation of HIFs is not uncommon: miR-155 [46], miR-424 [28], and the miR-17-92 cluster [30] are known to target HIF1α. Thus HIFs can induce the expression of miRNAs, and in turn, miRNAs can also target HIFs.

Our understanding of the regulation of HIF3α and its role in the hypoxic response is still rather limited. Reports in the literature are sometimes contradictory or difficult to compare as they focus on different HIF3α splice variants in different cell types. It is clear that HIF3α can be regulated at different levels. Transcription of HIF3α can be induced by HIF via HREs in the promoter region [5,7,11]. This probably accounts for the increased HIF3α mRNA expression upon hypoxia. In addition HIF3α can be regulated at the protein level. HIF1α and HIF2α contain an oxygen dependent degradation domain (ODDD), which harbors two conserved prolines that can be hydroxylated by prolyl hydroxylases (PHDs) in presence of oxygen. This marks the proteins for proteasomal degradation via the von Hippel-Lindau (VHL) E3 ubiquitin ligase complex. It was postulated that HIF3α, in contrast with HIF1α and HIF2α, is not regulated at the level of protein stability, since the longer HIF3α transcripts miss part of the ODDD and the shorter isoforms lack the entire ODDD. This renders them less prone to hydroxylation and subsequent proteasomal degradation [11]. However, other studies showed that the remaining proline residue in the ODDD of HIF3α transcripts is targeted by VHL and subjected to proteasomal degradation in an oxygen dependent manner [5,6]. Besides transcriptional regulation of HIF3α by HIF1 and oxygen dependent degradation of HIF3α proteins, we provide evidence for an additional layer of regulation of HIF3α, i.e. miRNAs that act on the translational level. These different mechanisms can complement one another in fine tuning HIF3α expression. This may be critical because of the versatile role HIF3α plays in the hypoxic response.

None of the HIF3α variants are likely to act as potent transcription factors, since they lack a C-terminal transactivation domain. However, they can have an effect on HRE-driven gene expression, depending on the level of HIFβ [5,8,10]. When HIFβ is not limiting, it will dimerize with HIF1α, HIF2α and HIF3α to induce a subset of hypoxia regulated genes. HIF1α/HIFβ and HIF2α/HIFβ complexes will associate with HRE sequences, while the HIF3α/HIFβ complexes likely bind a response element different from the canonical HRE, to maximize hypoxia induced gene expression [5]. When HIFβ is limiting, HIF3α will either compete with HIF1α and HIF2α for binding with HIFβ [8], or HIF3α will associate with HIF1α and HIF2α [5,10], both resulting in decreased capability to bind HRE sequences and diminished transcription of HIF target genes. As such, HIF3α splice variants will not act as global regulators of gene expressions, but may modulate specific genes in a cell type dependent manner [5,11].

## Conclusion

In conclusion, this study describes, in addition to miR-210-3p, a new panel of HRMs in hypoxic sarcoma cells. In turn, two of these HRMs, i.e. miR-210-3p and miR-485-5p, regulate HIF3α in a direct or indirect fashion, respectively. Fine-tuning of HIF3α expression is of great importance for the hypoxic response, since HIF3α affects HIF1/2-induced gene expression. Keeping these negative feedback loops under tight control enables the cells to adapt to hypoxic conditions. Deregulation of these mechanisms, e.g. by therapeutic modulation of levels of miRNAs that are important for the hypoxic response, may inhibit the adaptive potential of the cells, and reduce their resistance against radiation and systemic agents.

## Abbreviations

HIF: hypoxia inducible factor; HRE: hypoxia responsive elements; HRM: hypoxia responsive miRNA; LNA: locked nucleic acid; miRNA: microRNA; qPCR: quantitative polymerase chain reaction; STS: soft tissue sarcomas; 3’UTR: 3’untranslated region.

## Competing interests

The authors declare they have no competing interests.

## Authors’ contributions

CG, SS, JV, EW conceived the study. CG, EW, SS, RM, JV were involved in the design and coordination of the study. CG, PK, JR, MJ and NM performed qPCR, end-point PCR and Western blotting experiments. CG, WIJ, MJ carried out miRNA expression profiling CG, NM, EW participated in cloning experiments and luciferase reporter assays. CG, PK, WIJ, SS, RM, JV and EW drafted the manuscript. All authors read and approved the final version of the manuscript.

## Pre-publication history

The pre-publication history for this paper can be accessed here:

http://www.biomedcentral.com/1471-2407/14/429/prepub

## Supplementary Material

Additional file 1: Table S1 Primers used for PCR and cloning. (A) Primers used for end-point RT-PCRs of HIF3α 3’UTR fragments, resulting in amplification products of 212 bp (Fw/Rv1) and 313 bp (Fw/Rv2). Amplification of HPRT (product of 335 bp) was used as input control. (B) Primers used for cloning HIF3α-short and HIF3α-long 3’UTR constructs. (C) Primers used for site mutagenesis of predicted miR-485-5p and miR-210-3p binding sites in HIF3α-short 3’UTR constructs. **Table S2** Differentially expressed miRNAs (p<0.05) between cell lines that were cultured under hypoxic and normoxic conditions. P-values of two-sample t-test as well as fold change in miRNA expression and miRNA genomic locations are indicated. False Discovery Rate (FDR) for the top four miRNAs are: hsa-miR-185-3p (FDR 0.002893); hsa-miR-485-5p (FDR 0.004466); hsa-miR-216a-5p (FDR 0.068687) and hsa-miR-625-5p (FDR 0.112324). **Figure S1** Predicted 3’UTR target sites in HIF3α for miR-210-3p and miR-485-5p and the mutations that have been generated in the target site sequence where the miRNA seed sequence (bold) binds. The wild-type (WT) and mutated (mut) sites in HIF3α-short are shown. The vertical lines represent possible base pairing between miRNA and 3’UTR target site, and the x’s indicate abrogated base pairing where nucleotides are mutated (red). The resulting mutated 3’UTR fragments were cloned into the psiCHECK-2 luciferase reporter. Click here for file
