# Supplementary material for: MicroRNA response to hypoxic stress in soft tissue sarcoma cells: microRNA mediated regulation of HIF3α
Source: BMC Cancer. 2014 Jun 13;14:429. doi: 10.1186/1471-2407-14-429 (PMC4065608; doi:10.1186/1471-2407-14-429)
Supplement: Additional file 1: Table S1 — Primers used for PCR and cloning. (A) Primers used for end-point RT-PCRs of HIF3α 3’UTR fragments, resulting in amplification products of 212 bp (Fw/Rv1) and 313 bp (Fw/Rv2). Amplification of HPRT (product of 335 bp) was used as input control. (B) Primers used for cloning HIF3α-short and HIF3α-long 3’UTR constructs. (C) Primers used for site mutagenesis of predicted miR-485-5p and miR-210-3p binding sites in HIF3α-short 3’UTR constructs. Table S2 Differentially expressed miRNAs (p<0.05) between cell lines that were cultured under hypoxic and normoxic conditions. P-values of two-sample t-test as well as fold change in miRNA expression and miRNA genomic locations are indicated. False Discovery Rate (FDR) for the top four miRNAs are: hsa-miR-185-3p (FDR 0.002893); hsa-miR-485-5p (FDR 0.004466); hsa-miR-216a-5p (FDR 0.068687) and hsa-miR-625-5p (FDR 0.112324). Figure S1 Predicted 3’UTR target sites in HIF3α for miR-210-3p and miR-485-5p and the mutations that have been generated in the target site sequence where the miRNA seed sequence (bold) binds. The wild-type (WT) and mutated (mut) sites in HIF3α-short are shown. The vertical lines represent possible base pairing between miRNA and 3’UTR target site, and the x’s indicate abrogated base pairing where nucleotides are mutated (red). The resulting mutated 3’UTR fragments were cloned into the psiCHECK-2 luciferase reporter. [file 1471-2407-14-429-S1.doc]

**Additional file 1 Tables and Figure legends**

**Additional file 1:** Table S1: Primers used for PCR and cloning. (A) Primers used for end-point RT-PCRs of HIF3α 3’UTR fragments, resulting in amplification products of 212 bp (Fw/Rv1) and 313 bp (Fw/Rv2). Amplification of HPRT (product of 335 bp) was used as input control. (B) Primers used for cloning HIF3α-short and HIF3α-long 3’UTR constructs. (C) Primers used for site mutagenesis of predicted miR-485-5p and miR-210-3p binding sites in HIF3α-short 3’UTR constructs.

**Additional file 1:** Table S2: Differentially expressed miRNAs (p<0.05) between cell lines that were cultured under hypoxic and normoxic conditions. P-values of two-sample t-test as well as fold change in miRNA expression and miRNA genomic locations are indicated. False Discovery Rate (FDR) for the top four miRNAs are: hsa-miR-185-3p (FDR 0.002893); hsa-miR-485-5p (FDR 0.004466); hsa-miR-216a-5p (FDR 0.068687) and hsa-miR-625-5p (FDR 0.112324).

**Additional file 1:** Figure S1: Predicted 3’UTR target sites in HIF3α for miR-210-3p and miR-485-5p and the mutations that have been generated in the target site sequence where the miRNA seed sequence (bold) binds. The wild-type (WT) and mutated (mut) sites in HIF3α-short are shown. The vertical lines represent possible base pairing between miRNA and 3’UTR target site, and the x’s indicate abrogated base pairing where nucleotides are mutated (red). The resulting mutated 3’UTR fragments were cloned into the psiCHECK-2 luciferase reporter.

Supplemental Table 1A

| **Primer** | **Sequence** | **Product** |
| --- | --- | --- |
| HIF3α Fw1 | 5'-AGAACAATGATCCACGGGT-3' | 212 bp |
| HIF3α Rv1 | 5'-GCCTCAATCGGAAGTCAC-3' |
| HIF3α Fw2 | 5'-GCCTCACAGCTTCCAACT-3' | 313 bp |
| HIF3α Rv2 | 5'-TGGGGCACAGAGATTGTAG-3' |
| HPRT Fw | 5'-ATGGGAGGCCATCACATTG-3' | 336 bp |
| HPRT Rv | 5'-GGTCCTTTTCACCAGCAAG-3' |

Supplemental Table 1B

| **Primer**  **Cloning** | **Sequence** | **Product** |
| --- | --- | --- |
| Fw | 5’-GTCTCGAGCCGGCTCCTCTCCCCATCTG-3’ |  |
| Rv-short | 5’-GAGCGGCCGCAGACCACATTGGAGGTTG-3’ | 817 bp |
| Rv-long | 5’-GAGCGGCCGCGTGCCTACCAAGGTGAGGTCTTTAT-3’ | 3807 bp |

Supplemental Table 1C

| **Primer**  **Mutagenesis** | **Sequence** |
| --- | --- |
| HIF3a-485-5p Fw | 5’-CCTACTTCAGGGGCCGCGGCCCAGTTCCTCTGC-3’ |
| HIF3a-485-5p Rv | 5’-GCAGAGGAACTGGGCCGCGGCCCCTGAAGTAGG-3’ |
| HIF3a-210 Fw | 5’-CCACGCCGGCAGCCAAAGCTTAGGATGGGGGCG-3’ |
| HIF3a-210 Rv | 5’-CGCCCCCATCCTAAGCTTTGGCTGCCGGCGTGG-3’ |

Supplemental Table 2

|  | **MiRNA** | **Parametric p-value** | **Fold-change Up in Hypoxia** | **Fold-change Down in Hypoxia** | **Genomic Location** |
| --- | --- | --- | --- | --- | --- |
| 1 | hsa-miR-185-3p | 4,60E-06 | 1,89 |  | 22-q11.21 |
| 2 | hsa-miR-485-5p | 1,42E-05 | 2,61 |  | 14-q32.31 |
| 3 | hsa-miR-216a-5p | 0,0003276 | 1,91 |  | 2-p16.1 |
| 4 | hsa-miR-625-5p | 0,0007143 |  | 1,38 | 14-q23.3 |
| 5 | hsa-miR-553 | 0,0018115 |  | 1,33 | 1-p21.2 |
| 6 | hsa-miR-526a; hsa-miR-520c-5p; hsa-miR-518d-5p | 0,004425 |  | 1,19 | 19-q13.41 |
| 7 | hsa-miR-148b-3p | 0,0076947 |  | 1,18 | 12-q13.13 |
| 8 | hsa-miR-184 | 0,00935 |  | 1,24 | 15-q25.1 |
| 9 | hsa-miR-656-3p | 0,0094883 | 1,22 |  | 14-q32.31 |
| 10 | hsa-miR-488-3p | 0,0107997 |  | 1,24 | 1-q25.2 |
| 11 | hsa-miR-127-5p | 0,0110241 |  | 1,32 | 14-q32.31 |
| 12 | hsa-miR-541-3p | 0,0120157 |  | 1,26 | 14-q32.31 |
| 13 | hsa-miR-181b-5p | 0,0163786 |  | 1,23 | 1-q31.3; 9-q33.3 |
| 14 | hsa-miR-302b-5p | 0,0227309 |  | 1,23 | 4-q25 |
| 15 | hsa-miR-936 | 0,0258524 |  | 1,16 | 10-q25.1 |
| 16 | hsa-miR-944 | 0,0281864 |  | 1,24 | 3-q28 |
| 17 | hsa-let-7^e^-5p | 0,0291331 |  | 1,29 | 19-q13.33 |
| 18 | hsa-miR-149-5p | 0,0297993 | 1,31 |  | 2-q37.3 |
| 19 | hsa-miR-541-5p | 0,029902 | 1,30 |  | 14-q32.31 |
| 20 | hsa-miR-26a-1-3p | 0,0311843 | 1,16 |  | 3-p22.2 |
| 21 | hsa-miR-92b-5p | 0,034333 | 1,32 |  | 1-q22 |
| 22 | hsa-miR-501-5p | 0,0354958 | 1,23 |  | X-p11.23 |
| 23 | hsa-miR-342-5p | 0,0370505 |  | 1,19 | 14-q32.2 |
| 24 | hsa-miR-454-5p | 0,0399102 | 1,21 |  | 17-q22 |
| 25 | hsa-miR-191-5p | 0,0411142 | 1,19 |  | 3-p21.31 |
| 26 | hsa-miR-625-3p | 0,0424962 | 1,29 |  | 14-q23.3 |
| 27 | hsa-miR-373-3p | 0,0433468 | 1,18 |  | 19-q13.41 |
| 28 | hsa-miR-486-3p | 0,043349 |  | 1,28 | 8-p11.21 |
| 29 | hsa-miR-141-5p | 0,0469436 | 1,17 |  | 12-p13.31 |
| 30 | hsa-miR-25-3p | 0,0472339 | 1,18 |  | 7-q22.1 |
| 31 | hsa-miR-29b-1-5p | 0,0476345 |  | 1,22 | 7-q32.3 |
| 32 | hsa-miR-629-5p | 0,049196 |  | 1,20 | 15-q23 |

Supplemental Figure 1

5' ...CCACGCCGGCAGCCAACGCACAG... - HIF3α position 56-78 WT

││││ │ ││││││││

3'     AGUCGGCGACAGUG**UGCGUGU**C  - hsa-miR-210

││││ │ │X││XX││

5' ...CCACGCCGGCAGCCAA**A**GC**TT**AG... - HIF3α position 56-78 mut

5' ...CUCCUACUUCAGGGGCAGCCUCC... - HIF3α position 687-709 WT

│ │││ ││ │││││││
3'     CUUAAGUAGUGCCG**GUCGGAG**A - hsa-miR-485-5p

│ │││ ││ │X││XX│
5' ...CUCCUACUUCAGGGGC**C**GC**GG**CC... - HIF3α position 687-709 mut
